# Strategies and Behaviour Change Techniques to Optimise Sedentary Behaviour for People with Severe Asthma: A Nominal Group Technique

**DOI:** 10.3390/jcm15103879

**Published:** 2026-05-18

**Authors:** Paola D. Urroz Guerrero, Vanessa M. McDonald, Peter G. Gibson, Eleanor C. Majellano, Hayley Lewthwaite

**Affiliations:** 1 National Health and Medical Research Council Centre for Research Excellence in Treatable Traits, University of Newcastle, Newcastle, NSW 2305, Australiaeleanor.majellano@newcastle.edu.au (E.C.M.);; 2 School of Nursing and Midwifery, University of Newcastle, Newcastle, NSW 2308, Australia; 3 Breathing and Lung Health Program, Hunter Medical Research Institute, Newcastle, NSW 2305, Australia; 4 Department of Respiratory and Sleep Medicine, John Hunter Hospital, Newcastle, NSW 2305, Australia; 5 School of Medicine and Public Health, University of Newcastle, Newcastle, NSW 2308, Australia; 6 School of Biomedical Sciences and Pharmacy, University of Newcastle, Newcastle, NSW 2308, Australia

**Keywords:** severe asthma, sedentary behaviour, behaviour change, behaviour change techniques, nominal group technique

## Abstract

**Background:** While sedentary behaviour has been shown to be associated with adverse health outcomes for people with asthma, limited research has been done on how to improve sedentary behaviour in this population. We aimed to obtain key stakeholders’ perspectives on what is important to reduce sedentary time in people with severe asthma. **Methods:** Adults with severe asthma and their carers were invited to participate in a nominal group technique session. Participants volunteered strategies they considered important for optimising sedentary behaviour (reducing sedentary time and/or breaking up prolonged sedentary bouts). Following this, participants were instructed to identify the 10 most important strategies and rank these from most (10 points) to least (1 point) important. The 10 strategies that scored the highest proportion of the total possible score across all nominal group technique sessions were reported, and two independent researchers identified and deductively coded behaviour change techniques (BCTs) within these strategies. **Results:** Twenty participants attended one of five nominal group technique sessions. Severe asthma participants (*n* = 17) had a mean age (SD) of 69.9 (8.7) years, and self-reported spending a mean (SD) 7(3) hours per day sedentary. Carers (*n* = 3) had a mean (SD) age of 57.3 (21.0) years and self-reported a mean (SD) of 5 (2) hours of sedentary time per day. A total of 116 individual strategies were volunteered. With a weighted score of 8.3 (out of 10), “have a reminder or timer to minimise sedentary behaviour” was the highest weighted scored strategy. A total of 13 BCTs were coded to the list of the 10 highest weighted scored strategies. **Conclusions:** This study identified strategies important to people living with severe asthma and their carers for reducing time spent in sedentary behaviour. These strategies were coded to BCTs and can inform the design of future interventions to optimise sedentary behaviour. Future research should evaluate the effectiveness and feasibility of implementing these strategies.

## 1. Introduction

Sedentary behaviour is a term that entered public awareness in the early 2000s [1]. The term refers to a class of behaviour that is distinct from physical inactivity. A person can meet physical activity guidelines while still accumulating high levels of sedentary time. Physical inactivity refers to not meeting the recommended amount of moderate-to-vigorous physical activity (MVPA). Sedentary refers to high proportions of wake time spent in sedentary behaviour. Sedentary behaviours are those that: (1) have a low energy expenditure (≤1.5 metabolic equivalents of task [METs]) and are performed (2) in a sitting, reclining, or lying position (3) while awake [2]. Targeting sedentary behaviour has emerged as an important public health intervention, as time spent sedentary is an independent risk factor (even when accounting for time spent physically active) for all-cause mortality and incidence of cardiovascular disease, cancer, and type 2 diabetes [3,4,5].

Research is now emerging on the associations between sedentary behaviour and adverse asthma-specific health outcomes. More prolonged time spent sedentary has been shown to be associated with increased risk of hospitalisation for an asthma attack, worse asthma control, and reduced lung function and physical capacity [6,7].

This is of particular concern for people with severe asthma, who experience worse asthma outcomes and have a higher disease burden compared to those with mild to moderate asthma [8,9,10]. Although the biological mechanisms remain poorly understood, sedentary behaviour may influence asthma outcomes through pathways including systemic inflammation [11]. Developing interventions that target prolonged sedentary behaviour for people with severe asthma is therefore important and warranted, particularly since people with severe asthma have been shown to spend as much as 9 h per day sedentary [12].

To date, there have been no randomised controlled trials to explore the effectiveness of sedentary-behaviour-targeted interventions in people with severe asthma. Existing randomised controlled trials have primarily focused on the impact of physical activity interventions on both physical activity and sedentary behaviour outcomes. These have not resulted in meaningful reductions in sedentary behaviour [13]. Interventions that involve environmental restructuring, persuasion, education, self-monitoring, problem solving, and restructuring the social or physical environment have been shown to be promising techniques for reducing sedentary behaviour in the general population [14]. While lessons can be learned from the general population around how to reduce sedentary behaviour for people with asthma, there are also asthma-related barriers and facilitators to behaviour change that must be considered when designing interventions for this population, particularly for people with severe asthma. These include managing asthma symptoms, environmental exposures, and provision of support from healthcare professionals [13].

Sedentary behaviour interventions are considered complex, as it requires modifying established habits and routine. Recognising this complexity, the Medical Research Council framework for developing and evaluating complex interventions emphasises meaningful involvement of stakeholders as a key element for developing effective interventions [15]. This approach also emphasises the importance of identifying and developing theory as a key step in intervention design [15]. Stakeholder perspectives can provide an understanding of how and why people behave as they do and how behaviours can be modified (theory), which can then be used to design tailored interventions for specific populations. Previous research reported that people with severe asthma view prolonged sedentary behaviour negatively while considering it necessary for symptom management, have routines influenced by asthma control and responsibilities, and often misconceive that reducing sedentary time requires engaging in moderate-to-vigorous physical activity [16]. Building on this work can enable the strategic selection of strategies that are most likely to successfully reduce sedentary behaviour in people with severe asthma, ultimately increasing the probability of meaningful and sustainable behaviour change. This study, therefore, aimed to identify strategies to reduce time spent sedentary that were deemed important to people with severe asthma and their carers, informing the development of effective interventions that can be successfully translated into clinical practice.

## 2. Methods

### 2.1. Study Design

This study employed a modified nominal group technique (NGT), a validated semiquantitative method in clinical research for obtaining group consensus and priority setting among stakeholders [17,18]. The NGT was selected as the most appropriate methodology to achieve consensus over other methods due to its ability to achieve a quick consensus, and it provides a structured forum where generating ideas may be challenging [17]. The modification of the NGT involved conducting multiple independent NGT sessions across five focus groups, where strategies were generated and ranked within each group to achieve group-level consensus. People with severe asthma and their carers participated in face-to-face or virtual focus groups, with two to six participants required per group. Ethics approval was obtained from the Hunter New England Human Research Ethics Committee (2022/ETH02420). In accordance with Good Clinical Practice, informed written or electronic consent was obtained from each participant prior to data collection. The Consolidated Criteria for Reporting Qualitative Research was used to guide reporting [19].

### 2.2. Participant Recruitment and Selection

Adults (≥18 years) were included if they: (1) had confirmed evidence of variable airflow limitation within the past 10 years (bronchodilator response, airway hyperresponsiveness or peak flow variability) and (2a) were prescribed high-dose inhaled corticosteroids (>1000 μg beclomethasone equivalent) with a second controller and had uncontrolled asthma according to the European Respiratory Society/American Thoracic Society taskforce definition [20] or (2b) require a monoclonal antibody (mAb) therapy to control asthma or asthma that remained uncontrolled despite being on a mAb. Adults (≥18 years) who were providing unpaid care for ≥6 months (denoting informal carer) to a relative/friend who was recruited as part of the severe asthma group were also invited to participate. Severe asthma participants were recruited via the research database of the Department of Respiratory and Sleep Medicine’s Ambulatory care clinics at the John Hunter Hospital (Newcastle, Australia) or by their treating clinician/physician. Potential participants were identified by their treating clinician/physician and had their interest in the study ascertained before being contacted for participation by research personnel. Participants were selected using consecutive sampling.

### 2.3. Measures

All participants completed a structured interview over the telephone with a trained researcher, collecting data on demographics, health, and asthma. Movement behaviour was measured using the International Physical Activity Questionnaire (IPAQ) [21]. During the structured telephone interview, severe asthma participants were asked about their exacerbation history (previous 12 months) and completed additional validated questionnaires:

- The 6-item Asthma Control Questionnaire (ACQ6) to assess asthma control [22].
- Dyspnoea-12 (D-12) to provide an overall score (0–36) of daily life breathlessness, with a higher score indicating more severe breathlessness [23].
- Modified Medical Research Council Dyspnea Scale (mMRC) to assess functional breathlessness impact. A score ≥2 is considered as physically limiting breathlessness [24].

Clinical measures, including height, body mass and pre- and post-bronchodilator forced expiratory volume in one second (FEV_1_) and forced vital capacity (FVC), were obtained from medical records for severe asthma participants. Clinical measures were collected from medical records between March 2020 and April 2023. The Global Lung Index equations were used to calculate predicted values [25]. These data were collected to characterise and describe the population.

### 2.4. Focus Group Procedure

Focus groups were held in a private meeting room on the premises of the Hunter Medical Research Institute (Newcastle, NSW, Australia) or online using Zoom for a mean duration of 1 h and 37 min and were audio-recorded. Participants were compensated with a $50 gift card per session for time invested in contributing to the focus group. Present during the focus groups were the participants and two facilitators (PUG and HL). A semi-structured, open-ended interview guide was used by the primary and secondary facilitators who did not have an established role in providing clinical care to the participants. The primary facilitator was author PUG (PhD), who was a PhD candidate at the time of data collection and has experience in conducting qualitative research. The secondary facilitator was HL (PhD), a female post-doctoral fellow who also has previous experience conducting qualitative research.

**Figure 1:**
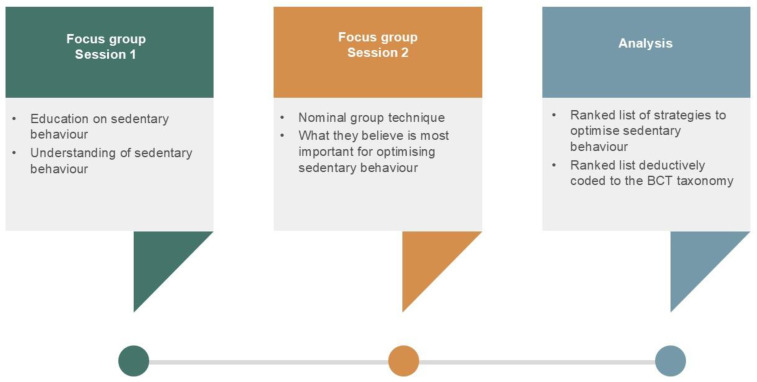
Phases of focus group discussions and the use of the nominal group technique.

Participants attended two sessions; each session was two weeks apart (Figure 1). The first session involved following a semi-structured interview guide to gauge participants’ understanding of sedentary behaviour. A standardised educational presentation was delivered to all participants by PUG on sedentary behaviour (10 to 15 min) during session one. Topics that were covered during the presentation included defining what activities are considered sedentary behaviour, outlining the national recommendations for physical activity and sedentary behaviour, and the difference between active/inactive and sedentary/non-sedentary. Participants also completed activities to confirm their ability to correctly identify behaviours that were sedentary.

The second session involved a structured NGT (Supplementary Materials). This was conducted in four components:

- The session facilitators invited participants to individually brainstorm and silently write ideas for strategies that would help people with severe asthma to reduce sedentary time and/or break up prolonged sedentary bouts;
- In a group discussion, the session facilitators went around the group and collated and recorded the ideas of each participant, verbatim;
- The facilitators went through each listed idea and provided an opportunity for discussion and clarification of the idea;
- Participants were asked to vote independently to prioritise the ideas, from a collated list, according to what they believe is most important for optimising sedentary behaviour. They were asked to select the 10 most important ideas and to rank them from most important (10 marks) to least important (1 mark).

### 2.5. Data Generation and Analysis

A descriptive summary analysis was completed to characterise the sample using SPSS statistics version 28 (IBM, Armonk, NY, USA). The results of the NGT for each focus group were collated to calculate the overall sum of ranks. A weighted score was calculated, with the sum of scores for each strategy divided by the total possible score multiplied by 10. The total possible score varied depending on the number of participants in each focus group and represented the maximum score achievable if all participants allocated the highest score of 10 to a strategy. For example, in a focus group with six participants, the maximum possible score would be 60 (6 × 10), and a strategy receiving 30 points would have a weighted score of (30/60) × 10 = 5. The top 10 ranked strategies were deductively coded to the BCT [26] using NVivo 12 Pro (QSR International, Melbourne, VIC, Australia). The behaviour change taxonomy is a structured system for classifying and organising 93 distinct “active ingredients” within behaviour change interventions. Two team members trained in coding for the behaviour change taxonomy v1 independently reviewed and coded the strategies, giving consideration to the definitions of each of the BCTs [26]. Strategies could be coded to more than one BCT where multiple behavioural components were present within a single strategy. Any discrepancies between coders were discussed and resolved through consensus. All elements in the interpretation of the data were discussed with the authorship team, ensuring that a consensus on the final analysis was reached.

## 3. Results

Five nominal group sessions were conducted with a total of 20 participants, with 3 to 5 participants attending each focus group. The nominal group session had a mean (SD) duration of 97.4(24.4) min. Table 1 presents participant characteristics.

### 3.1. Severe Asthma Participants

Participants with severe asthma (*n* = 17) were predominantly female (71%) with a mean (SD) age of 69.9 (8.7) years, ACQ-6 of 1.7 (1.3), and mMRC of 2.3 (1.6). Most (84%) participants were prescribed mAb therapy, and more than half (53%) were not currently employed or were retired. People with severe asthma self-reported spending a mean (SD) of 7 (3) h per day sedentary.

### 3.2. Carers of Severe Asthma Participants

Three carers participated in the focus groups (parent/child, *n* = 1; spouse/partner *n* = 2). Carers were younger than the severe asthma participants with a mean (SD) age of 57.3 (21.0) years and most were not working or retired (67%). They self-reported a mean (SD) of 5 (2) hours of sedentary time per day.

### 3.3. Nominal Group Technique

A total of 116 individual strategies were collated from the NGT. Of the 116 strategies, 98 items were selected by participants as part of their top 10 list of strategies important to optimise sedentary behaviour. The highest weighted scored strategy was “Have a reminder or timer to minimise sedentary behaviour” with a weighted score of 8.3 (out of 10). Table 2 presents the highest 10 ranked strategies according to their weighted score.

### 3.4. Behaviour Change Techniques

The 10 highest ranked strategies were coded to the BCT taxonomy. Inter-rater agreement across behaviour change technique coding ranged from 79% to 99%, indicating high agreement between coders. Table 3 lists the BCTs that were identified and the frequency of coding for each listed BCT. A total of 13 BCTs were coded to the list of top 10 ranked strategies, with the most frequently coded BCT being “Behaviour substitution”. The most common BCT categories included “Goals and planning” (four strategies), “Antecedents” (four strategies) and “Repetition and substitution” (two strategies). Supplementary Table S2 presents the final agreed behaviour change techniques identified within each top ranked strategy.

## 4. Discussion

This novel study reports strategies and techniques considered important by people with severe asthma and their carers to optimise sedentary behaviour. These strategies address the needs of people with asthma and may help to overcome asthma-specific barriers to optimising sedentary behaviour not experienced by the general population. The prioritised list of strategies highlight three main messages: (1) there was an apparent misperception that structured exercise was required to replace sedentary behaviour; (2) strategies identified as important were mostly in line with what is known from the general population; (3) there is, however, consideration of approaches within these strategies that were asthma-specific, including setting goals within reduced physical capacity limits and breathlessness management. These findings can inform potential effective intervention components targeting sedentary behaviour in people with severe asthma, providing a foundation for future research.

A change in sedentary time necessitates an equal and opposite change in one or more other behaviours over the day (i.e., LPA, MVPA and/or sleep) [27,28]. In our study, people with severe asthma and their carers prioritised replacing time spent sedentary with time spent participating in MVPA, particularly in the form of structured exercise (Table 2). Displacing the proportion of the day spent in sedentary behaviour to MVPA in people with severe asthma is likely to lead to beneficial effects, and doing 4–5 times the recommended amount of MVPA per week (approximately 38 min of vigorous physical activity or 76 min of moderate physical per day) can offset the detrimental effects of sedentary behaviour [29,30]. However, people with severe asthma experience multidimensional physical activity barriers that remain difficult to overcome [13]. Therefore, replacing a meaningful proportion of their sedentary behaviour with MVPA is not feasible and, instead, replacing sedentary time with LPA is more achievable to obtain health benefits. 

The desire to replace sedentary behaviour with MVPA may be attributed to a misperception that MVPA is the only meaningful health behaviour. The strategies identified through the NGT suggest that, despite the educational overview provided, participants often interpreted optimising sedentary behaviour as replacing sedentary time with structured physical activity. This highlights that improving understanding of what sedentary behaviour is and how it can be modified may itself be an important component of future interventions. Therefore, education around sedentary behaviour may be a necessary first step when designing interventions aimed at optimising sedentary behaviour in people with severe asthma. In a large 15-year follow-up cohort study, substituting even 30 min of sedentary time with LPA was associated with reduced all-cause mortality and cardiovascular disease mortality [31]. Providing evidence to people with severe asthma surrounding the benefits of replacing sedentary time with LPA will be important to facilitate behaviour change.

Goal setting is a commonly used BCT in sedentary behaviour interventions across different populations [14]. In this study, participants identified goals that were focused on the achievement they desired to reach as a result of optimising sedentary behaviour (e.g., increasing the number of steps and/or distance walked per day, Table 2) [32,33]. Participants also recognised that goals needed to be achievable and measurable with self-monitoring. Given the heterogeneous nature of severe asthma, shared decision making may be important when setting individualised goals that account for functional ability and available resources [34]. When developing tailored plans with people with severe asthma, the “when”, “where” and “how” will need to be specified for each individual based on their identified sedentary behaviour habits [35].

Sedentary behaviour is often driven by established habits [36]. In this study, people with severe asthma and their carers recognise this and elected strategies that reshape these habits. In unfamiliar situations, behaviour is regulated by making a deliberate decision, whereas, in familiar settings, behaviour is regulated by habitual or automatic processes [36,37]. For example, in our study, participants prioritised “walking while talking on the phone” as a strategy, which suggests that talking on the phone is a “familiar” sedentary behaviour situation and therefore elected to break this habit and replace it with another (i.e., standing or walking). Using prompts or cues to change behaviour was seen as important by participants in this study. Cue–behaviour associations is an approach that facilitates the transformation of behaviour change from a deliberate decision into an automatic or non-conscious process [38,39]. Using cues or prompts as a behaviour change technique to optimise sedentary behaviour shows promising results in desk-based workers [40], however, does not appear to be a commonly used BCT in clinical populations [41]. Future sedentary behaviour interventions for people with severe asthma should consider tailoring the intervention to individual preferences to reduce the burden associated with prompts and cues [20].

Different types of physical and social environmental factors are associated with sedentary behaviour [42]. Participants in our study prioritised strategies that involved being outdoors (e.g., walk a certain distance around the block/units or be outdoors to engage in outdoor-related activities, Table 2). This supports findings of older adults who spend only 5% of their sedentary time outdoors and 70% at home [43]. However, certain environmental conditions are known to trigger asthma symptoms, such as cold, dry air, air pollution and pollen. Therefore, prompting or encouraging people with severe asthma to be outdoors as a strategy to reduce sedentary behaviour may need to be tailored to each individual’s environmental triggers. To counterbalance, providing strategies for reducing sedentary behaviour when indoors is also likely needed when being impacted by outdoor weather and air quality. In this study, people with severe asthma and their carers also recognised that the social context of sedentary behaviour matters. This finding is supported by evidence indicating that social support is associated with a decrease in sedentary behaviour [44]. Additionally, social support has been identified as a key behaviour change in technique in digital-supported sedentary behaviour interventions [45].

The strategies highlighted by people with severe asthma and their carers often included asthma-tailored approaches. Using self-monitoring devices and setting goals can help increase awareness and provide motivation to improve sedentary behaviour. However, for people with severe asthma, it is important to set realistic and achievable goals that consider disease-specific barriers. Limitations to exercise capacity, breathlessness or the risk of an exacerbation were key concerns identified by our participants (e.g., not to walk too far away, in case you cannot get back; learn how to control breathlessness, Table 2). Sedentary time is associated with exercise capacity in people with severe asthma [7]. Additionally, multimorbidity is common in people with severe asthma and may further shape sedentary behaviour patterns and the strategies individuals perceive as feasible [46]. Co-existing chronic conditions such as obesity, osteoporosis, and metabolic disease can contribute to functional limitations that influence activity behaviours. In the present cohort, obesity was prevalent (mean BMI 32.1 kg/m^2^), which may have influenced both sedentary behaviour levels and the types of strategies prioritised by participants. This warrants consideration of the functional ability of people with severe asthma when setting goals and recommending tasks or activities to optimise sedentary behaviour patterns. For example, breaking down a large task into small achievable steps may be necessary. The burden of breathlessness in people with severe asthma is high, with one study reporting 53% of people with severe asthma experiencing physically limiting breathlessness [47]. People with severe asthma have previously reported breathlessness as their primary problem of importance [48], a theme that continues in our findings, where “learning how to control breathlessness” is a highly-rated strategy. When delivering sedentary behaviour interventions for people with severe asthma, breathlessness self-management and energy conservation strategies are likely important.

This study is novel as we report prioritised strategies to optimise sedentary behaviour in people with severe asthma, identified by people with severe asthma and their carers. The strength of this study includes the use of the NGT as a methodology to elicit the perspective of key stakeholders, providing an unbiased interpretation of their perspectives. This study also includes carers of people with severe asthma, recognising the important role they play in the lives of people living with severe asthma [49]. However, the study is not without some limitations. The generalisability of our findings is limited by the demographic characteristics of the sample. Participants were predominantly older, retired and Caucasian, which may have influenced the types of strategies generated. For example, several strategies reflected routines more common among retired individuals (e.g., morning routines or walking around the block). These strategies may therefore not fully translate to younger individuals with severe asthma who are working or have different daily schedules. Although the inclusion of carers provides valuable exploratory insights into the support dynamics of severe asthma, this group was small (*n* = 3) and remains underrepresented. As such, the findings primarily reflect the perspectives of people living with severe asthma, and the views of carers should be interpreted with caution.

Due to the absence of comorbidity data we are unable to determine the impact of comorbidities on the study outcomes. Sedentary time was assessed using the IPAQ, which has known limitations when estimating sedentary behaviour compared with device-based measures such as accelerometery. Therefore, the self-reported sedentary time reported in this study may underestimate actual sedentary behaviour levels among participants. Another limitation was that there was only one round of the NGT process. A second round of voting on a compiled list of volunteered strategies generated by other focus groups might have influenced participants’ preferences. However, the sample size achieved in this study is considered appropriate for the NGT methodology [50]. This study does not evaluate the effectiveness of implementing these strategies; therefore, we cannot determine whether the identified approaches effectively reduce sedentary behaviour or improve asthma outcomes. However, efficacy testing was beyond the scope of this research.

## 5. Conclusions

This study presents a prioritised list of strategies to optimise sedentary behaviour in people with severe asthma by key stakeholders. This led to the identification of BCTs that may be important for designing a sedentary-behaviour-focused intervention, increasing the likelihood of developing an acceptable intervention for people with severe asthma. People with severe asthma and their carers identified strategies well-known from the general population such as goal-setting, social support and changing the environment. Strategies that address asthma-specific barriers were also identified such as setting goals within their physical capacity and managing breathlessness, highlighting factors clinicians may need to consider when supporting people with severe asthma to modify their movement behaviours. These findings also highlight the importance of clinicians discussing sedentary behaviour with people with severe asthma, as patients may not always recognise the distinction between sedentary behaviour and physical inactivity. These strategies should now be evaluated with randomised controlled trials to assess their effectiveness, feasibility and potential for implementation.

## Figures and Tables

**Table 1:** Demographics and characteristics of participants.

	Severe Asthma (*n* = 17)	Carers (*n* = 3)
Demographics		
Age (years)	69.9 ± 8.7 (53.3–82.2) ^#^	57.3 ± 21.0 (33.2–71.4) ^#^
Sex (female)	12 (71%)	2 (67%)
Ethnicity		
Caucasian	16 (94%)	2 (67%)
Aboriginal or Torres Strait Islander	1 (6%)	-
African	-	1 (33%)
Living arrangements		
Living with spouse/partner	8 (47%)	2 (67%)
Living with family	5 (30%)	1 (33%)
Living alone	4 (24%)	-
Employment or study status		
Not working for other reasons/retired	9 (53%)	2 (67%)
Not studying or working due to health reasons	4 (24%)	-
Working full-time or part-time	3 (18%)	1 (33%)
Domestic duties	1 (6%)	-
Movement behaviours		
Walking (MET-min per week)	480 (0, 1360)	2400 (578, 2400)
Moderate-intensity physical activity (MET-min per week)	160 (0, 3120)	1680 (1200, 1680)
Vigorous-intensity physical activity (MET-min per week)	0 (0, 1200)	720 (720, 720)
Sedentary time (hours per day)	7 ± 3	5 ± 2
Medications		
Monoclonal antibody therapy	14 (84%)	-
ICS daily dose, beclomethasone equivalent (mcg)	1588 ± 982	-
LAMA	6 (32%)	-
Macrolides	5 (26%)	-
Maintenance OCS	4 (21%)	-
Clinical measures		
BMI (kg/m^2^)	32.1 ± 6.1	-
Post-bronchodilator FEV_1_ (% predicted)	74 ± 25	-
Post-bronchodilator FEV_1_/FVC	0.63 ± 0.17	-
ACQ-6 (average score)	1.7 ± 1.3	-
Patient-reported measures		
Dyspnoea-12 (total score)	9.6 ± 8.9	-
mMRC (score)	2.3 ± 1.6	-
Exacerbations in the previous 12 months	2 (0, 3)	-

Unless otherwise stated, data are reported as mean ± standard deviation or median (interquartile range) or number (proportion). ^#^ (range); MET, metabolic equivalent of task; ICS, inhaled corticosteroids; LAMA, long-acting muscarinic antagonists; OCS, oral corticosteroids; BMI, body mass index; FEV_1_, forced expired volume in 1 s; FVC, forced vital capacity; ACQ, asthma control questionnaire; D12, Dyspnoea-12; mMRC, modified Medical Research Council.

**Table 2:** Weighted highest ranked strategies to optimise sedentary behaviour according to people with severe asthma and their carers.

Strategy		Weighted Score (out of 10)
1	Have a reminder or timer to minimise sedentary behaviour	8.3
2	Regular exercise, to replace sedentary behaviour	7.8
3	Walk as often as I can, use fitness watch to achieve goals and as incentive for movement and sit less	7.4
4	Have a goal to walk a certain distance around the block/units, or walk where it is flat, to progress—not to walk too far away, in case you can’t get back.	6.8
5	Be outdoors to engage in outdoor-related activities	6.5
6	Learn how to control breathlessness	6.5
7	Set a daily step target (e.g., 10,000/day)	6.3
8	Have the assistance of family members or others to do activities	6.0
9	Walk around when on the phone	6.0
10	Make a routine to do things in the morning—get up and going	5.8

Weighted score = (sum of score/total possible score) × 10.

**Table 3:** Frequencies of behaviour change techniques coded to top 10 ranked strategies to optimise sedentary behaviour by people with severe asthma and their carers.

BCT		Definition	Times Coded
1	8.2 Behaviour substitution	Prompt substitution of the unwanted behaviour with a wanted or neutral behaviour	4
2	7.1 Prompts or cues	Introduce or define environmental or social stimulus with the purpose of prompting or cueing the behaviour. The prompt or cue would normally occur at the time or place of performance	3
3	1.4 Action planning	Prompt detailed planning of performance of the behaviour (must include at least one of context, frequency, duration and intensity).	3
4	1.3 Goal setting (outcome)	Set or agree on a goal defined in terms of a positive outcome of wanted behaviour	3
5	8.4 Habit reversal	Prompt rehearsal and repetition of an alternative behaviour to replace an unwanted habitual behaviour	3
6	1.2 Problem solving	Analyse, or prompt the person to analyse, factors influencing the behaviour and generate or select strategies that include overcoming barriers and/or increasing facilitators	2
7	12.1 Restructuring the physical environment	Change, or advise to change the physical environment to facilitate performance of the wanted behaviour or create barriers to the unwanted behaviour	2
8	3.1 Social support (unspecified)	Advise on, arrange or provide social support (e.g., from friends, relatives) or non-contingent praise or reward for performance of the behaviour.	2
9	12.3 Avoidance/reducing exposure to cues for the behaviour	Advise on how to avoid exposure to specific social and contextual/physical cues for the behaviour, including changing daily or weekly routines	2
10	2.3 Self-monitoring of behaviour	Establish a method for the person to monitor and record their behaviour(s) as part of a behaviour change strategy	1
11	1.1 Goal setting (behaviour)	Set or agree on a goal defined in terms of the behaviour to be achieved	1
12	12.6 Body Changes	Alter body structure, functioning or support directly to facilitate behaviour change	1
13	12.2 Restructuring the social environment	Change, or advise to change the social Environment in order to facilitate performance of the wanted behaviour or create barriers to the unwanted behaviour	1

## Data Availability

All data generated or analysed during this study are included in this published article.

## Supplementary Materials

The following supporting information can be downloaded at: https://www.mdpi.com/article/10.3390/jcm15103879/s1, Table S1: Nominal Group Technique Structure; Table S2: Behaviour change techniques coded to the highest ranked strategies.
