# Supplementary material for: Strategies and Behaviour Change Techniques to Optimise Sedentary Behaviour for People with Severe Asthma: A Nominal Group Technique
Source: J Clin Med. 2026 May 18;15(10):3879. doi: 10.3390/jcm15103879 (PMC13207782; doi:10.3390/jcm15103879)
Supplement: Supplementary file 1 [file jcm-15-03879-s001.zip › jcm-4235522-supplementary.pdf]

Supplementary Materials

Table S1. Nominal Group Technique Structure

Component	Instructions
Component 1: The session moderator will ask participants to individually brainstorm and silently write ideas on strategies that would help people with severe asthma to be less sedentary.	We would like you to write down the strategies you do or you think are important to reduce time spent being sedentary or interrupt long sitting bouts. We ask that you do this silently, and that you don't discuss your ideas with one another as we are interested in your individual ideas. We will allow up to 15 minutes for this step.
Component 2: In a group discussion, the session moderator will go around the table and collate and record the ideas of each participant. These will be recorded on the flip chart/screen for everyone to see.	We are going to go around the table where everyone can take turns to read out their ideas without any elaboration or justification. We will do this later. If you come up with other ideas as we go around the room, we encourage that you share this even though it may not be on your list. I will ask that each person to share one of their ideas and then we move onto the next person. You always have the option to 'pass' on your turn. We will continue to go around until no new ideas have been presented or until we reach the end of the allocated time for this step which is 15 minutes.
Component 3: The moderator will go through each listed idea and allow for an opportunity of discussion and clarification of the idea.	We will now go through and discuss each idea on the list and give a chance for you to clarify any ideas presented. If you don't agree with any idea presented, we can give you a chance to show your preference in the next discussion section.
Component 4: Participants will be asked to vote privately to prioritise the ideas according to what they believe is most important for optimising sedentary behaviour. They will be asked to select the 10 most important ideas and to rank them from most important (10 marks) to least important (1 mark). The votes will be tallied to	Looking at the list that we have come-up with, we would like everyone to individually write down their top 10 ideas and to rank them from most important which will score 10 points to least important which will score 1 point. We will then tally the rankings and identify which ideas are most preferred by the group.

show the highest rated idea by the group.	

Table S2: Behaviour change techniques coded to the highest ranked strategies

Strategy	Final agreed BCT(s)
Have a reminder or timer to minimise sedentary behaviour	• Prompts or cues
Regular exercise to replace sedentary behaviour	• Behaviour substitution<br>• Habit reversal
Walk as often as possible, use a fitness watch to achieve goals and as an incentive for movement and sit less	• Goal setting (behaviour)<br>• Behaviour substitution<br>• Self-monitoring of behaviour
Have a goal to walk a certain distance around the block/units to progress – not to walk too far away in case you cannot get back	• Goal setting (outcome)<br>• Problem solving Action planning
Be outdoors to engage in outdoor-related activities	• Prompts or cues<br>• Restructuring the physical environment
Learn how to control breathlessness	• Problem solving<br>• Body changes
Set a daily step target (e.g., 10,000/day)	• Goal setting (outcome)
Have the assistance of family members or others to do activities	• Social support (unspecified)
Walk around when on the phone	• Behaviour substitution<br>• Habit reversal<br>• Prompts or cues
Make a routine to do things in the morning – get up and going	• Action planning
